# A Monte Carlo Simulation Approach to Optimizing Capacity in a High-Volume Congenital Heart Pediatric Surgical Center

**DOI:** 10.3389/frhs.2021.787358

**Published:** 2022-02-10

**Authors:** Eleni G. Elia, Shirley Ge, Lisa Bergersen, Ravi R. Thiagarajan, Jason Thornton, Lynn A. Sleeper, Francis Fynn-Thompson, Derek Mathieu, Peta M. A. Alexander

**Affiliations:** ^1^Department of Cardiology, Boston Children's Hospital, Boston, MA, United States; ^2^Department of Pediatrics, Harvard Medical School, Boston, MA, United States; ^3^Department of Cardiac Surgery, Boston Children's Hospital, Boston, MA, United States; ^4^Department of Surgery, Harvard Medical School, Boston, MA, United States

**Keywords:** Monte Carlo simulation (MC), pediatric surgical center, elective surgeries scheduling, high volume hospital, congenital heart disease

## Abstract

**Importance:**

Elective surgeries are primarily scheduled according to surgeon availability with less consideration of patients' postoperative cardiac intensive care unit (CICU) length of stay. Furthermore, the CICU census can exhibit a high rate of variation in which the CICU is operating at over-capacity, resulting in admission delays and cancellations; or under-capacity, resulting in underutilized labor and overhead expenditures.

**Objective:**

To identify strategies to reduce variation in CICU occupancy levels and avoid late patient surgery cancellation.

**Design:**

Monte Carlo simulation study of the daily and weekly CICU census at Boston Children's Hospital Heart Center. Data on all surgical admissions to and discharges from the CICU at Boston Children's Hospital between September 1, 2009 and November 2019 were included to obtain the distribution of length of stay for the simulation study. The available data allows us to model realistic length of stay samples that include short and extended lengths of stay.

**Main Outcomes:**

Annual number of patient surgical cancellations and change in average daily census.

**Results:**

We demonstrate that the models of strategic scheduling would result in up to 57% reduction in patient surgical cancellations, increase the historically low Monday census and decrease the historically higher late-mid-week (Wednesday and Thursday) censuses in our center.

**Conclusions and Relevance:**

Use of strategic scheduling may improve surgical capacity and reduce the number of annual cancellations. The reduction of peaks and valleys in the weekly census corresponds to a reduction of underutilization and overutilization of the system.

## Keypoints

**Question:** Can the occupancy of a cardiac intensive care unit (CICU) at a high-volume congenital heart center be modeled in a computer simulation to identify optimal elective surgical scheduling practices?
**Findings:** Our CICU weekly occupancy was modeled in a Monte Carlo simulation using historical data as inputs. Scheduling patients with expected hospital stays of 1–2 days earlier in the week on Monday and Tuesday reduced variation in CICU census and the number of annual surgical cancellations.
**Meaning:** Strategic scheduling of surgeries may reduce CICU census variation, increase the number of patients served, and minimize cancellations.

## Introduction

Post-operative recovery following congenital heart disease (CHD) surgery requires specialized care in a cardiac intensive care unit (CICU). CICU beds are a scarce resource and require careful planning, allocation, and management (1–3). In intensive care settings, quality of care is generally measured by low mortality, and metrics for waste include admission delays, surgical cancellations, extended length of stay (LOS), and the need for increased care due to complications. Since the cost of many aspects of critical care are fixed, e.g., room and board fees including nursing salaries, serving more patients at a time in the ICU significantly increases hospital revenue and ultimately reduces the cost of care per patient. Consequently, CICUs often operate at or near capacity, which result in cancellations of scheduled elective surgeries and delays when providing urgent non-surgical critical care (2).

Postoperative CICU LOS in CHD patients can be accurately predicted pre-operatively through clinical factors and the surgical procedure type that is to be performed (4). Scheduling elective surgeries with consideration of expected CICU LOS can potentially maximize CICU capacity while minimizing waste. Elective surgeries are usually scheduled based only on preferences of patients and surgeons, and without consideration of downstream limitations such as CICU capacity. The CICU census (i.e., actual bed occupancy) can exhibit a high rate of variation in which a CICU may operate over capacity, resulting in admission delays and cancellations, or significantly under-capacity, resulting in wasted labor and overhead expenditures. Reducing variability in scheduled surgical caseloads can potentially relieve strain on CICU capacity because most diversions of care from the CICU occur during the unit's busiest periods (5). In this study, we simulated the weekly CICU census at our institution with Monte Carlo methods to demonstrate occupancy patterns and describe scheduling strategies to reduce variation in CICU capacity.

We hypothesized that an anticipatory simulation model, where surgeries with short LOS are scheduled earlier in the week and those with long LOS are scheduled later in the week, would reduce the number of surgical cancellations and variation in daily CICU census. By scheduling shorter stay patients on Mondays and Tuesdays, we are hoping to increase discharges later in the week and prevent the naturally occurring higher occupancy rates on Wednesday through Friday. Subsequently, by scheduling longer stay patients Wednesday through Friday, we may decrease weekend discharges and increase utilization of beds when performing elective procedures. The aim of our study was to identify optimal capacity management of our single-center CICU through Monte Carlo simulation of cardiac surgical scheduling. Additionally, we performed a sensitivity analysis to explore the impacts of day of admission, additional OR availability, and CICU LOS on the daily CICU census.

## Methods

### Study Design

We incorporated Boston Children's Hospital Heart Center admission and discharge data over a 10 year period to obtain the empirical distribution of CICU LOS to sample in the simulation study (namely to obtain the location and scale parameters of the distribution). This CICU LOS distribution and queuing theory principles informed Monte Carlo simulation of 500 years of data (i.e. 500 iterations) in order to model occupancy of the CICU under a variety of settings. We then simulated a 1-year time period of admissions with the same distribution of length of stay to model CICU occupancy rates. Local institutional review board approval was obtained.

### Setting

Boston Children's Hospital Heart Center is a quaternary referral center that provides surgical and non-surgical care to local, national and international patients with congenital and acquired heart disease. The high-volume center is served by a 31-bed CICU and adjoining 46-bed cardiology ward. Elective cardiac surgeries are only scheduled on weekdays. The Heart Center has 3 operating rooms (OR) devoted to cardiac surgery and shares 2 ORs with the orthopedic surgery program. The shared ORs are used weekly by the Heart Center on Tuesdays and Wednesdays. In total, there is capacity to perform 7 cardiac surgical procedures daily on Tuesday and Wednesday, and 5 surgical procedures daily on Monday, Thursday, and Friday.

### Participants

All admissions to and discharge from the Boston Children's Hospital CICU between September 1, 2009 and November 2019 informed the analysis. In total, there were 14,526 admissions. Descriptive data of the informing surgical population is included in recent reports of our program (4, 6). In brief, these admissions are typically comprised of 19% neonates (<30 days); 32% infants (30 days to <1year), 41% children (1 to <18 years) and 9% over age 18 years, with surgical complexity according to The Society of Thoracic Surgeons-European Association for Cardio-Thoracic Surgery (STAT) Category 1 25%, Category 2 29%, Category 3 15%, Category 4 25% and Category 5 5%. The distribution of length of stay for the cohort used in this report was median 2.0 days with an interquartile range of 1.0–6.0 days (Supplementary Figure 1).

Medical admissions were neither electively scheduled or predictable. All simulated admissions were controlled through the OR. As medical admissions historically make up 20% of Boston Children's Hospital CICU bed days, for the purposes of anticipatory modeling, we reduced the simulated threshold of our ICU capacity by 20%.

### Simulation Design

The primary outcome of this study was simulated occupancy of the CICU, with simulated anticipated CICU LOS, day of surgery, and number of available OR slots. Between September 1, 2009 and November 30, 2019, there were 14,526 admissions to the CICU. We used the LOS data from this cohort of patients to design the simulation. The LOS data for all patients admitted to the CICU in the study period followed a log-normal distribution (location = 1.114 and scale parameter = 1.085). This distribution is well suited for LOS, as it is able to accommodate patients with extended LOS (>30 days) (7–10). The LOS for each simulation run was sampled from this log-normal distribution.

Monte Carlo simulation, written in the R language (version 3.5.3) (11), uses the weekly arrival rate of patients as input into the simulation. The weekly arrival rate was calculated using Little's Law from queueing theory:

$$\begin{array}{l} \lambda \;=\frac{L}{W} \end{array}$$

where λ is the average number of patients arriving in the CICU each day, *L* is the average number of resources in the queueing system, and in this simulation study is set to the maximum CICU occupancy (25 beds), and *W* is the mean LOS from historical data (6.56 days) (12). The maximum CICU occupancy was set to 25 beds, instead of 31 beds, because approximately 20% (6 beds) of the CICU are occupied by non-surgical patients. The weekly arrival rate is obtained by multiplying λ by 5, for 5 operating days. In each simulation experiment, patient demand is randomly sampled from the interval [*a, b*] with lower bound “*a*” representing the minimum number of ORs per week (or the minimum number of weekly surgical patients) and the upper bound “*b*” representing the weekly number of patients who actually arrive, whilst guarding that the number of patients arriving for 52 weeks would not exceed the total annual surgical availability. The minimum number of weekly surgical availability was set to 8, as this reflects the average minimum number of weekly surgical procedures performed at our institution.

In order to provide recommendations for capacity planning, several scenarios with different simulation parameters were tested to estimate the expected number of cancellations. For each scenario, 500 replications of 365 days were run to simulate a year. At each replication, the throughput of patients was first equilibrated with a 30-day “warm-up” period, which was not included in the total year simulation. The choice of 30 days of warm-up is the typical length of running the event simulation prior to data collection to achieve steady-state performance measures. The warm-up allowed for the unit to achieve a natural level of occupancy without artificially selecting a starting point. Without it, the CICU occupancy would start at 0 patients, which would result in an underestimate of annual cancellations.

Next, the daily CICU census was calculated by taking the difference between admitted and discharged number of patients up to each date in a year. The census from the 500 replications of each scenario was aggregated and summary measures, including mean and standard deviation, were captured. The average number of cancellations across replications was calculated by the number of times the daily census exceeded the number of beds in the system.

### Models and Scenarios Tested

**Figure 1:**
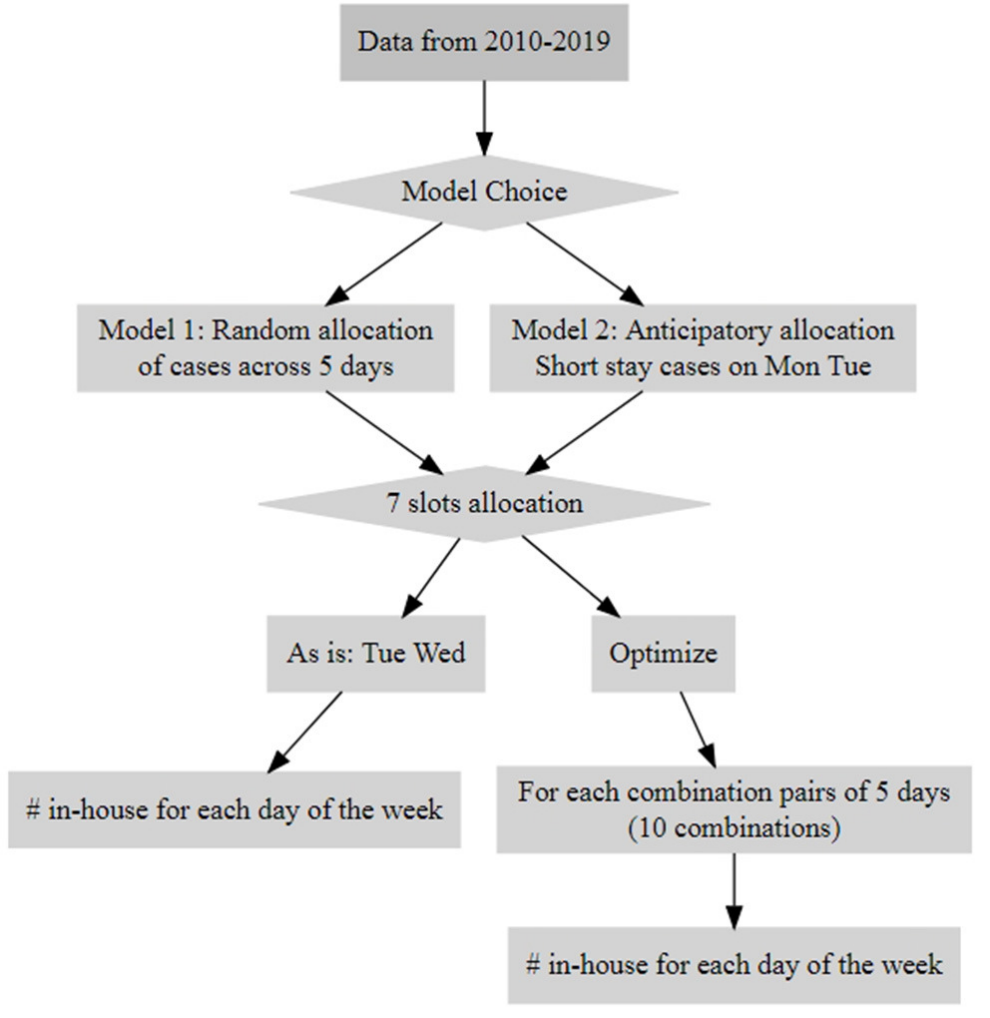
Flow chart of simulation decisions for Monte Carlo included models.

We conducted Monte Carlo simulation experiments that evaluated two models (Figure 1, Data Supplement). In the first model, assignment for the day of surgery was chosen randomly as a proxy for the current scheduling process; this model will be referred to as the “random model”. The second model was a surgical allocation model which will be referred to as the “anticipatory model”. The anticipatory model scheduled cases with short LOS (1–2 d) earlier in the week on Monday and Tuesday. It sought to minimize occupancy variance and reduce cancellations by clustering short-stay patients earlier in the week so that they could be discharged before the end of the week. Consequently, long-stay patients would cluster later in the week, with anticipated stay in the CICU over the weekend when elective surgeries typically do not occur (Table 1).

**Table 1:** Simulation model definitions.

**Model**	**Scheduling type**	**Additional slots allocation**
Model 1A	Random	Current OR allocation
Model 1B	Random	Mon and Fri (optimal pair of days for Random scheduling)
Model 2A	Anticipatory	Current OR allocation
Model 2B	Anticipatory	Wed and Thu (Optimal pair of days for Anticipatory scheduling)

For both models, the optimal arrangement for additional surgical scheduling currently available in the system (2 additional available surgical procedures in ORs [total = 7] on Tuesday and Wednesday) was also explored, testing each combination pair of 5 operating days (10 combinations total for each pair of days from Monday to Friday that the additional 2 ORs can be allocated to) (Supplementary Tables A1, A2). To identify the optimal allocation, we obtained the differences of the minimum and maximum statistics (mean and standard deviation) of average daily census for each pair of 10 days at 76% occupancy. The pair of days with the smallest differences for mean and standard deviation was identified as the optimal pair to which to allocate the additional surgical procedures (Table 1).

The experiments also varied the levels of occupancy (76, 86, and 90%).

## Results

**Figure 2:**
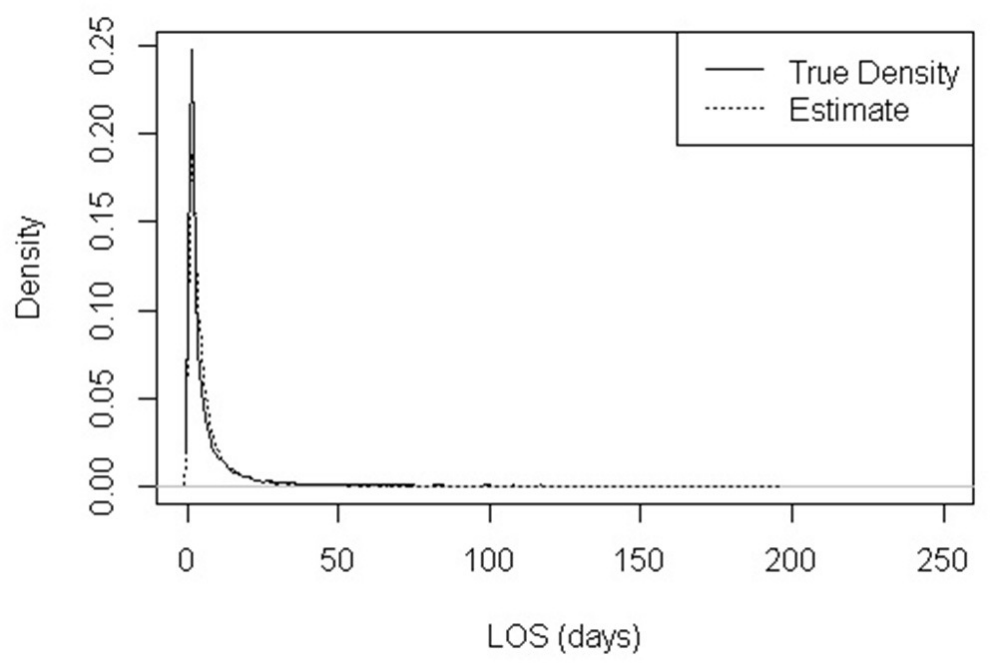
Observed distribution of CICU length of stay (LOS) and Lognormal-modeled distribution (location = 1.114 and scale parameter = 1.085).

The density distribution of CICU LOS based on data acquired from the study period and the log-normal density distribution of CICU LOS from Monte Carlo simulation samples are shown in Figure 2. The estimated density distribution closely aligns with the actual data. Both show that CICU LOS is positively skewed; a majority of patients stay in the CICU for a few days while a small percentage of patients have extremely long LOS, with 0.45% exceeding 100 days.

### Optimized Operative Availability (5 Available ORs Daily Plus 2 Additional Procedures Twice per Week)

Supplementary Tables A1, A2 present the determination of the optimal OR procedure allocation associated with the additional available OR for the random and anticipatory models, respectively. For the random model, Monday and Friday were the optimal pair. For the anticipatory model, the optimal days were Wednesday and Thursday.

### Annual Cancellations

Table 2 compares the number of cancellations per year for the two models and their optimal OR allocations. In the current OR set up (Models 1 and 2), with 7 procedures available on Tuesday and Wednesday, the anticipatory model yields 13–14% fewer cancellations than the random model. When the random model is performed with additional ORs on Monday and Friday, there is a 24–32% reduction in cancellations compared to the random model as is (Models 1A and 1B). The anticipatory model with 7 procedures available on Wednesday and Thursday (Model 2B) resulted in the greatest reduction of cancellations, yielding 45–57% fewer cancellations than the random model with the current OR schedule (Model 1A).

**Table 2:** Annual numbers and percentages of cancellations based on random scheduling (Model 1) and anticipatory scheduling (Model 2).

**Occupancy**	**7 slots on days**	**Number of cancellations**		**Annual average census**
		**Model 1**	**Model 2**	
76%	Tuesday and Wednesday	8.4 (0.7%)	8.3 (0.7%)	1,115
86%		25.4 (2.0%)	22.2 (1.8%)	1,262
90%		35.3 (2.7%)	30.4 (2.3%)	1,316
76%	Monday and Friday	5.7 (0.4%)	5.4 (0.4%)	1,115
86%		18.2 (1.5%)	17.4 (1.4%)	1,262
90%		26.7 (2.1%)	24.5 (1.9%)	1,316
76%	Wednesday and Thursday	9.1 (0.8%)	3.6 (0.2%)	1,113
86%		25.2 (2.0%)	12.6 (1.0%)	1,261
90%		35.9 (2.7%)	19.3 (1.4%)	1,314

*Additional surgical procedures allocated on days: Tuesday and Wednesday (current OR allocation, Model 1), Monday and Friday (Optimal pair of days for random scheduling, Model 1B), Wednesday and Thursday (Optimal pair of days for anticipatory scheduling, Model 2B)*.

### Census Weekly Variation

**Figure 3:**
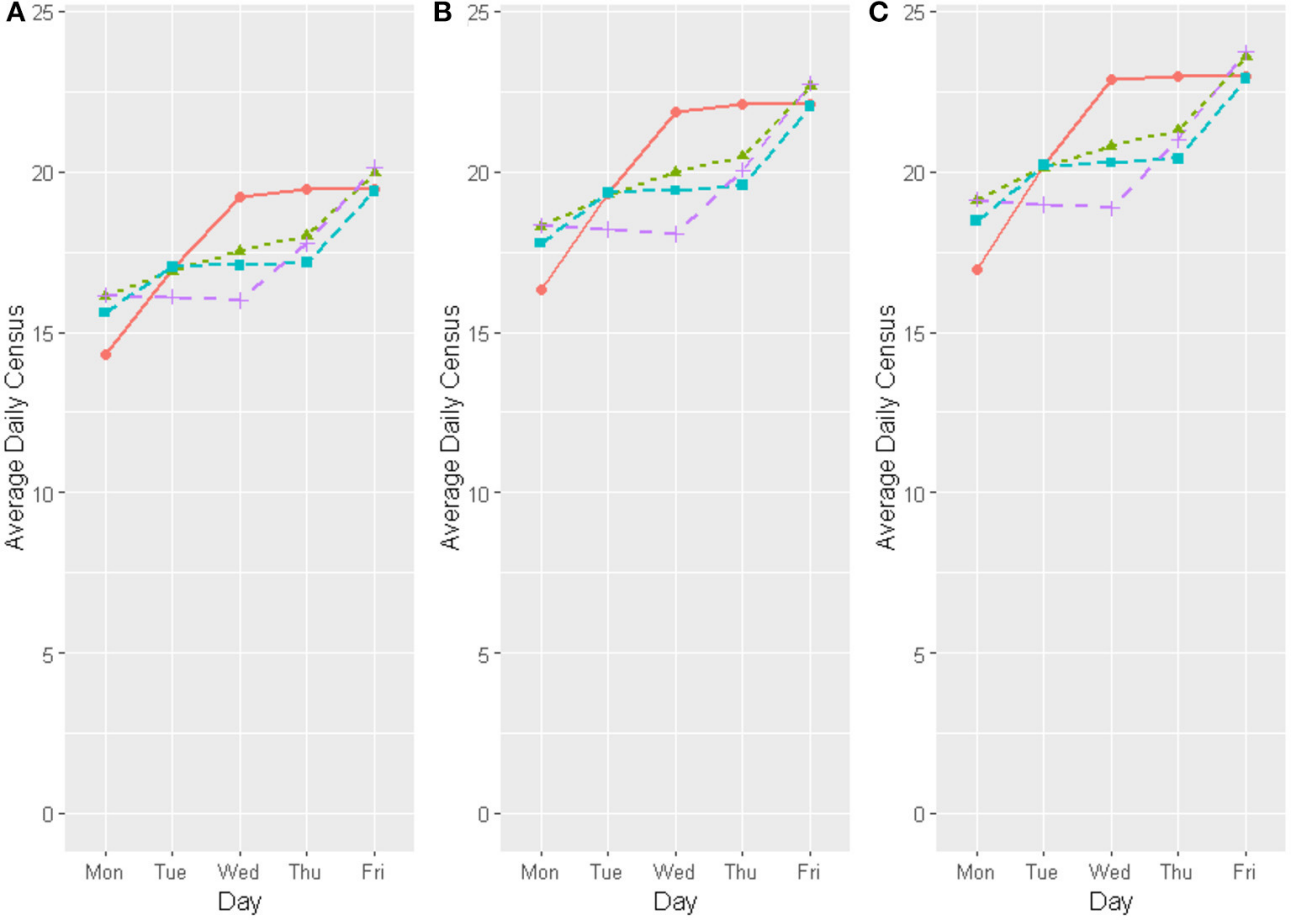
Comparison of daily average for CICU census in different capacities [**(A)** 76%, **(B)** 86%, and **(C)** 90% occupancy]; for different Model choices as follows: Model (1A) Current operating room allocation with random scheduling, Model (1B) Optimal day allocation of the additional operating room slots with random scheduling, Model (2A) Current operating room allocation with anticipatory scheduling, and Model (2B) Optimal day allocation of the additional operating room slots with anticipatory scheduling. Model 1A, Red line; Model 1B, Green dotted line; Model 2A, Light blue dashed line; Model 2B, Fuscia dashed line.

Assessing the average census per weekday (Figure 3), each of the tested scenarios show improved CICU capacity compared with the random model with the original OR allocation (Model 1). Though all the scenarios, including the random model as is, predict high CICU occupancy on Fridays, the scenarios with the anticipatory model and the random model with optimized OR days display a lower mean number of days at which the CICU is at high occupancy. Therefore, the alternative scenarios (Models 1B, 2A, and 2B) are more likely to have fewer canceled surgeries.

The anticipatory and random allocation model with optimized allocation of the 2 additional procedures (Models 1B and 2B) decreased the high points on the weekly census and increased the low points; the models showed an increase in the historically low Monday census and a decrease in the historically higher censuses on Wednesday and Thursday (Figure 3 and Table 3). A decreased weekly variation was also observed (Table 3). This translates to decreases in overutilization and underutilization, smoothing the weekly schedule and reducing peaks and valleys in capacity.

**Table 3:** Average daily census for current operating room allocation or optimal operating room allocation for the random and anticipatory models.

**Day**	**Average census**			
	**Model 1A**	**Model 1B**	**Model 2A**	**Model 2B**
Monday	14.31	16.12	15.60	16.14
Tuesday	16.94	16.91	17.04	16.10
Wednesday	19.24	17.53	17.09	15.98
Thursday	19.43	18.00	17.17	17.75
Friday	19.45	19.96	19.40	20.13
**SD**	2.26	1.44	1.36	1.78

*SD is the standard deviation of the weekly average census*.

## Discussion

This study demonstrated the use of a mathematical simulation approach to determine the optimal surgical scheduling parameters for reducing variation in CICU occupancy in a large quaternary pediatric institution. We found that an anticipatory scheduling model may reduce the number of cancellations by 13–14% and result in fewer high occupancy days, but with slightly higher variation in daily CICU census than the currently used model based on patient and surgeon preferences. When the anticipatory model was enhanced by utilizing specific days of the week that optimized additional OR availability, it yielded the least number of annual cancellations and the least variation in daily CICU census among the four scenarios tested.

The simulations showed that adopting an anticipatory scheduling approach and changing the allocation of OR availability to have 7 procedures on Wednesday and Friday and 5 procedures on Monday, Tuesday, and Thursday would considerably reduce the number of cancellations and the variation in the CICU daily census. Implementation of this approach is predicated on the ability to predict patient CICU LOS prior to surgical scheduling. We have previously demonstrated that pre-operative characteristics of this patient population can be used to predict post-operative CICU LOS with operationally adequate accuracy, and have incorporated these models into local capacity predictions (4, 6). In short, we derived Congenital Heart Surgical Stay categories, which closely approximate STAT Categories, to identify those patients with a short anticipated CICU course of 1–2 days (category 1–2), compared to those with a longer anticipated LOS (categories 3–5) (4). Additional considerations may also impact implementation, for example, in our center the additional OR availability comes from a shared space with the orthopedic surgery program. Changing OR allocation would require coordination with the orthopedics department. Our models may provide justification for changes in OR allocation by demonstrating a positive impact on patient care, staffing, and hospital finances. However, the impact on workflow by optimizing allocation of shared resources for one department may have deleterious consequences on resource allocation in another area; therefore, broader evaluation is required.

By understanding patterns of CICU occupancy, clinicians and administrators can identify and shift care models to efficiently manage available CICU beds. In general, beds are managed by crude historical estimates of need. This simulation of CICU census can help institutions make more informed decisions regarding optimal scheduling which will in turn, improve quality of care and reduce waste. High ICU occupancy rates have been shown to be associated with increased mortality, adverse events, and cancellations, while low occupancy leads to unnecessary expenses (13, 14).

This simulation approach can also aid in long-term planning of CICU operations, such as calculating staffing needs by using a projected daily census. Our Heart Center will be expanding into a new building in 2022 to 6 ORs, 48 CICU beds, and 48 inpatient rooms in the step-down unit. Modeling the new CICU with the building's parameters can inform staffing needs and help develop strategies to ramp up operations without producing excessive waste, delaying patient procedures, or impacting quality of care.

Furthermore, this simulation study could be readily tailored to other institutions and ICUs. It incorporates data from the daily ICU census and the parameters of the design could be targeted to unique institutional structural or programmatic requirements. Another advantage of our approach is that it considers the downstream capacity limitations brought on by ICU occupancy. Other simulation studies do not always consider the link between the throughput of surgeries and capacity of the ICU. They model the number of ORs needed or the ICU occupancy independently of the center's surgical practices (14, 15). Further studies with our simulation model can investigate the impact of specific clinical characteristics, such as patient age, or seasons (e.g., at the Heart Center, more pediatric elective surgeries are scheduled during summer vacation than during other months) on CICU occupancy. The simulation study can also be extended to use time series analysis to identify changes in daily census over time and capture dynamic patterns over time in specific centers.

Our study presents several limitations. First, it does not account for developments in treatments, clinical practices, or changes in cardiac surgeon staffing over the 10-year period of retrospective data that may impact recovery time, complications, or typical lengths of stay. In the simulation, however, the likelihood that an admission was sampled did not depend on the date of the admission. At our institution, there have not been any systematic scheduling procedures; surgeries have always been scheduled according to patient acuity, surgeon preferences and OR availability. Thus, we did not believe that changes in treatments or practices would significantly impact the accuracy of the model. Further, our results are generalizable to other centers with a patient cohort that has a distribution of length of stay that is similar to that used in this study; otherwise, the recommended scheduling may differ from that presented here. Lastly, the historical data did not exclude non-surgical patients, so the model sampled patients who underwent surgery in the OR and patients who did not. The medical patients only account for 10–15% of admissions to the CICU and the CICU LOS distribution of surgical patients has a wide range, so including the medical patients likely did not significantly skew the data. In future studies, the patients can be distinguished by medical and surgical admission to further explore CICU occupancy patterns.

## Conclusions

We developed a Monte Carlo simulation of CICU occupancy that models the weekly and daily censuses of the CICU from historical data. The results of the simulation experiments can help identify strategies to reduce the number of surgical cancellations and optimize ICU capacity at a high-volume center. By scheduling patients with an expected short stay to be early in the week and patients with an expected long stay to be later in the week, the variation in weekly CICU census decreased, thus reducing heterogeneity in weekly occupancy. Reducing CICU census variation also resulted in an increase in the annual number of patients served, while minimizing cancellations under the same system constraints. Similar simulation models can be created and applied to non-cardiac ICU and other types of units in order to optimize resource use and improve efficiency and cost-effectiveness of care.

## Data Availability Statement

The raw data supporting the conclusions of this article will be made available by the authors, without undue reservation.

## Ethics Statement

Local institutional review board approval was obtained. Written informed consent from the participants' legal guardian/next of kin was not required to participate in this study in accordance with the national legislation and the institutional requirements.

## Author Contributions

DM, PA, and LB: conceived the research idea. EE: designed the simulation and undertook the analyses and interpretation with the supporting supervision and feedback from DM and LS. EE and SG: drafted the paper and revised it following comments from all of the authors. All authors contributed to the interpretation of the results and critically revised the manuscript. All authors approved the final version of the manuscript.

## Conflict of Interest

The authors declare that the research was conducted in the absence of any commercial or financial relationships that could be construed as a potential conflict of interest.

## Publisher's Note

All claims expressed in this article are solely those of the authors and do not necessarily represent those of their affiliated organizations, or those of the publisher, the editors and the reviewers. Any product that may be evaluated in this article, or claim that may be made by its manufacturer, is not guaranteed or endorsed by the publisher.
