# Supplementary material for: A Monte Carlo Simulation Approach to Optimizing Capacity in a High-Volume Congenital Heart Pediatric Surgical Center
Source: Front Health Serv. 2022 Feb 10;1:787358. doi: 10.3389/frhs.2021.787358 (PMC10012657; doi:10.3389/frhs.2021.787358)

**DATA SUPPLEMENT:** A Monte Carlo Simulation Approach to Optimizing Capacity in a High-Volume Congenital Heart Pediatric Surgical Center

**AUTHORS:** Eleni G. Elia, MSc, PhD^1♦^; Shirley Ge, BS^1♦^; Lisa Bergersen, MD, MPH^1,2^; Ravi R. Thiagarajan, MBBS, MPH^1,2^; Jason Thornton, DNP, RN, NE-BC, CPHQ^1,2^; Lynn A. Sleeper, ScD^1,2^; Francis Fynn-Thompson, MD^3^; Derek Mathieu, MBA^1^; Peta M. A. Alexander MBBS^1,2*^

Institutional Affiliation: Department of Cardiology, Boston Children’s Hospital^1^; Department of Pediatrics, Harvard Medical School^2^; Department of Cardiac Surgery, Boston Children’s Hospital; Department of Surgery, Harvard Medical School^3^.

^♦^These authors contributed equally to this work.

Supplemental File includes:

1. SUPPLEMENTAL FILE 1. Pseudocode of Monte Carlo simulation study of CICU Length of Stay
2. Supplemental Figure: Observed Distribution of CICU Patients’ Length of Stay informing the Monte Carlo simulation.

SUPPLEMENTAL FILE 1. Pseudocode of Monte Carlo simulation study of CICU Length of Stay


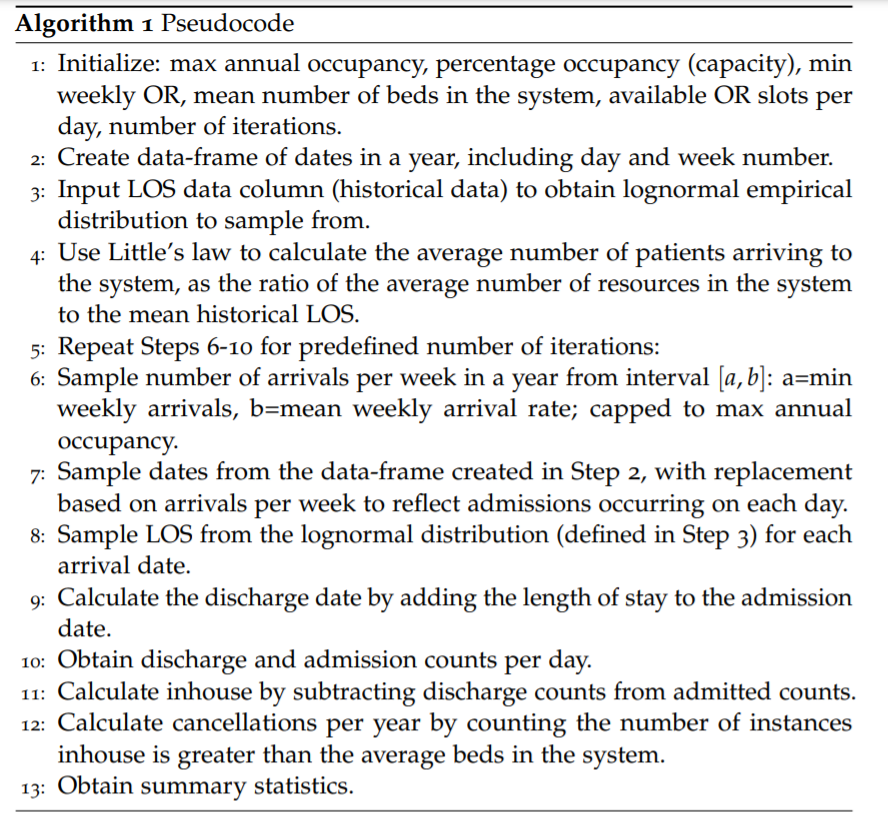


Supplemental Figure: Observed Distribution of CICU Patients’ Length of Stay informing the Monte Carlo simulation. N=14,526.


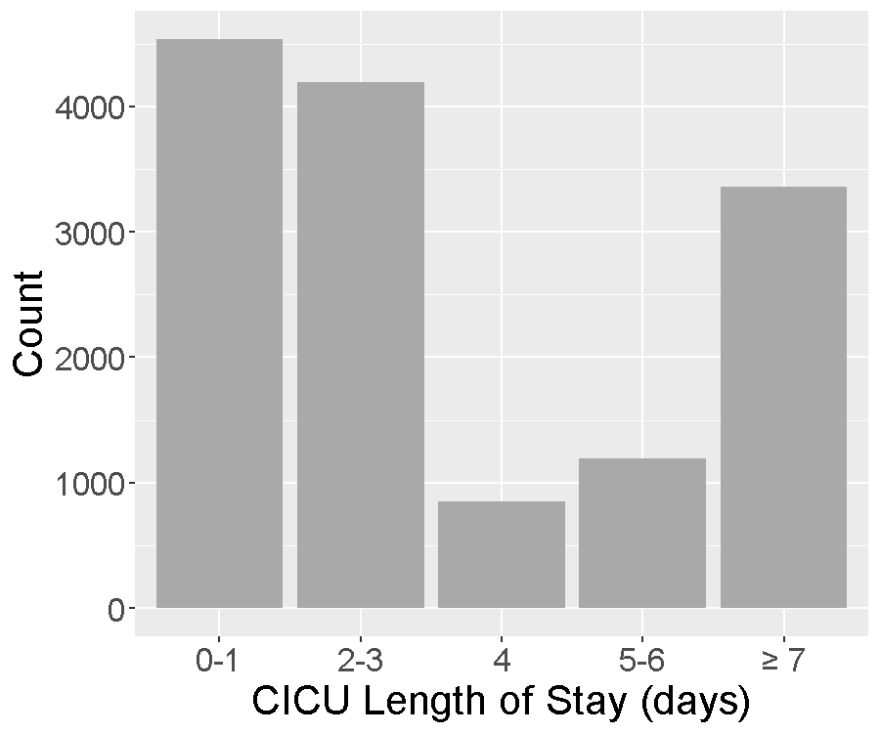

Supplement: Supplementary file 1 [file Data_Sheet_1.docx]
