# Supplementary figures and images for: A Monte Carlo Simulation Approach to Optimizing Capacity in a High-Volume Congenital Heart Pediatric Surgical Center
Source: Front Health Serv. 2022 Feb 10;1:787358. doi: 10.3389/frhs.2021.787358 (PMC10012657; doi:10.3389/frhs.2021.787358)

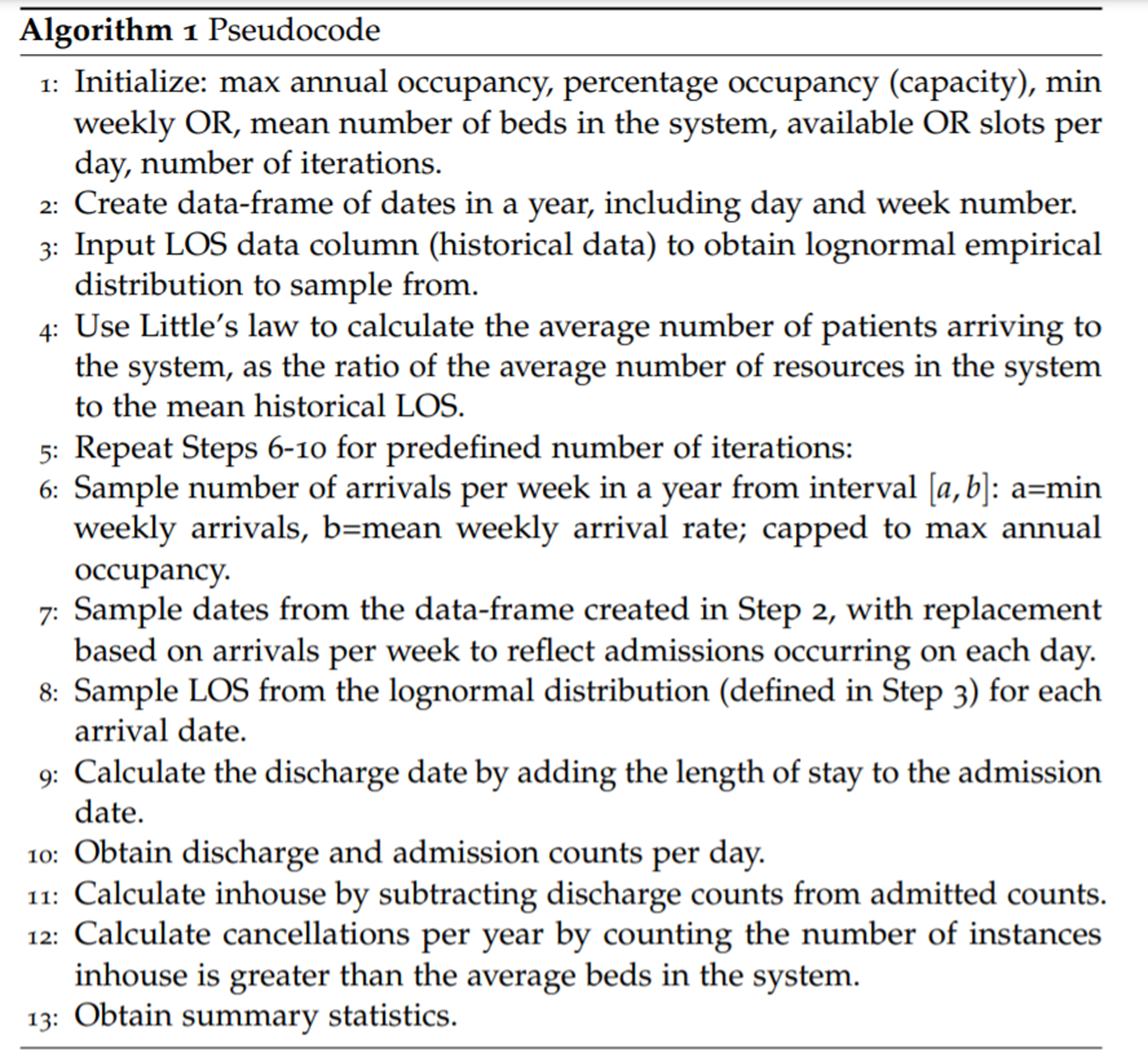

Supplement: Supplementary file 2 [file Image_1.png]

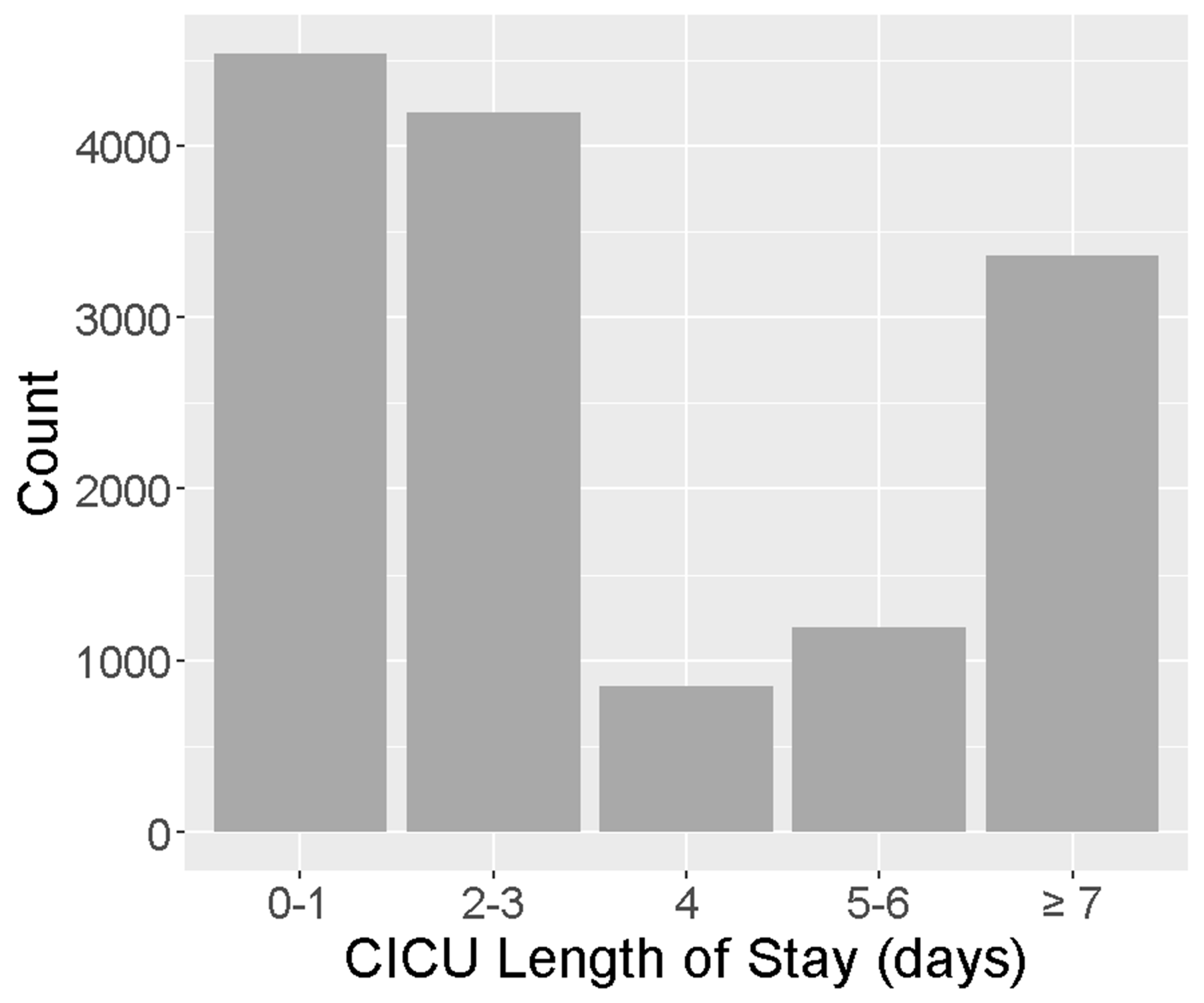

Supplement: Supplementary file 3 [file Image_2.tiff]
